# mTORC1-Sch9 regulates hydrogen sulfide production through the transsulfuration pathway

**DOI:** 10.18632/aging.102327

**Published:** 2019-10-03

**Authors:** Zhou Lyu, Xuejie Gao, Weiyan Wang, Jinye Dang, Li Yang, Mengli Yan, Shah Arman Ali, Yang Liu, Binghua Liu, Meng Yu, Linfang Du, Ke Liu

**Affiliations:** 1 Key Laboratory of Bio-Resources and Eco-Environment of Ministry of Education, College of Life Science, Sichuan University, Chengdu 610064, Sichuan, China; 2 Laboratory of Molecular Biology, College of Medicine, Chengdu University, Chengdu 610106, Sichuan, China

**Keywords:** hydrogen sulfide, Sch9, mTORC1, cystathionine gamma-lyase, cystathionine beta-synthase

## Abstract

Endogenous hydrogen sulfide mediates anti-aging benefits of dietary restriction (DR). However, it is unclear how H_2_S production is regulated by pathways related to DR. Due to the importance of mTORC1 pathway in DR, we investigated the effects of Sch9, a yeast homolog of mammalian S6K1 and a major substrate of mTORC1 on H_2_S production in yeast *Saccharomyces cerevisiae*. We found that inhibition of the mTORC1-Sch9 pathway by *SCH9* deletion, rapamycin or myriocin treatment resulted in a dramatic decrease in H_2_S production. Although deficiency of *SCH9* did not alter the intracellular level of methionine, the intracellular level of cysteine increased in *Δsch9* cells. The expression of *CYS3* and *CYS4*, two transsulfuration pathway genes encoding cystathionine gamma-lyase (CGL) and cystathionine beta-synthase (CBS), were also decreased under mTORC1-Sch9 inhibition. Overexpression of *CYS3* or *CYS4* in *Δsch9* cells or WT cells treated with rapamycin rescued the deficiency of H_2_S production. Finally, we also observed a reduction in H_2_S production and lowering of both mRNA and protein levels of CGL and CBS in cultured human cells treated with rapamycin to reduce mTORC1 pathway activity. Thus, our findings reveal a probably conserved mechanism in which H_2_S production by the transsulfuration pathway is regulated by mTORC1-Sch9 signaling.

## INTRODUCTION

The roles of Hydrogen sulfide (H_2_S) as a gaseous signal transmitter has been well-appreciated in the last two decades [1–4]. There are many signaling pathways in a range of organisms, from yeast to human, that are regulated by H_2_S including cell death, the cell cycle, autophagy, inflammation, aging and oxidative stress. Physiologically, H_2_S plays an important role in protecting the nervous system and the cardiovascular system of animals [5, 6].

Endogenous production of H_2_S is mainly catalyzed by four enzymes involved in cysteine metabolism including cystathionine gamma-lyase (CGL), cystathionine beta-synthase (CBS), cysteine aminotransferase (CAT) and 3-mercaptopyruvate sulfurtransferase (3MST). The production of H_2_S can be controlled by the expression of these enzymes, the bioavailability of their substrates, and enzyme activity modulating factors [3]. Therefore, the regulation of H_2_S production is complicated and more studies are required to clarify how it is controlled under physiological or pathological conditions.

Recently it was suggested that endogenous H_2_S production due to sulfide amino acids restriction is essential for anti-aging benefits of dietary restriction (DR) [7]. Similarly, methionine restriction extends eukaryotic life span probably through a mechanism involved in H_2_S production as well [8]. Mechanistic target of rapamycin complex 1 (mTORC1) pathway also plays a key role in the anti-aging effects of DR [9, 10]. Inhibiting mTORC1 pathway by rapamycin treatment or by deletion of down-stream signaling components such as *SCH9*, an homologue of mammalian S6K1 in *Saccharomyces cerevisiae* and one of direct substrates of mTORC1, mimics DR and provides anti-aging benefits [11–13]. However, it is unknown if the mTORC1 pathway regulates H_2_S production even though it mediates at least some effects of DR. Since the mTORC1-Sch9 pathway in yeasts responds to DR [14, 15] and is required for protein synthesis and amino acid metabolism [16–18], we sought to determine if mTORC1-Sch9 regulates H_2_S production via sulfide amino acids metabolism.

## RESULTS

### Inhibiting mTORC1-Sch9 inhibits H_2_S production

**Figure 1:**
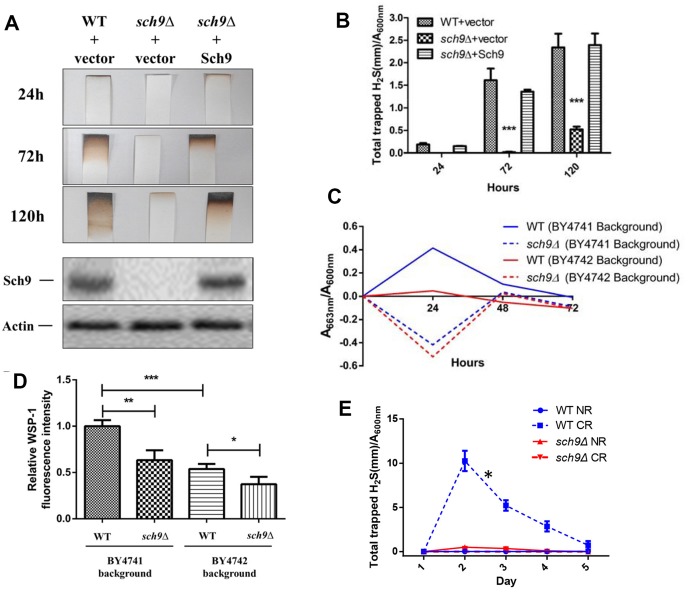
**Deletion of *SCH9* decreased H_2_S production in different yeast strains.** (**A**) WT and *Δsch9* cells in the TB50a background were transformed with pRS316-*SCH9* or empty vector and inoculated into 1L of SDC medium at initial OD_600nm_=0.005. H_2_S production was monitored using lead acetate strips at indicated times (Upper 3 panels) after inoculation. The level of Sch9 protein and actin loading control were determined by Western blotting as shown in the lower 2 panels. (**B**) Millimeters of darkening of the lead acetate strips inserted into the headspace of the culture flask shown in panel A normalized by OD_600nm_. (**C**) Methylene blue assays of H_2_S produced by WT and *Δsch9* cells in BY4741 or BY4742 background. Note that there is spontaneous oxidation of methylene blue when H_2_S is absent which gave negative readings for methylene reduction (red and blue dash lines). (**D**) Intracellular H_2_S production in WT and *Δsch9* cells in BY4741 or BY4742 background monitored by H_2_S fluorescent with probe WSP-1. (* p<0.05; ** p<0.01; *** p<0.005). (**E**) H_2_S production by WT and *Δsch9* cells in BY4742 background assayed by using lead acetate strips which were replaced every 24 hours under caloric restriction conditions (CR, medium containing 0.5% glucose) or no restriction (NR, medium containing 2% glucose).

Sch9 is a direct substrate of yeast mTORC1 and depletion of *SCH9* extends yeast lifespan through mechanisms shared with lifespan extension by calorie restriction (CR) [13, 16]. Since H_2_S mediates the benefits of CR, we first compared H_2_S production in *Δsch9* mutant cells to WT cells. While WT cells released measurable amounts of H_2_S, *Δsch9* cells produce barely detectable amounts of H_2_S (Figure 1A and 1B). H_2_S production was recovered if a functional *SCH9* gene was added back to the mutant cells (Figure 1A and 1B), thus, showing that Sch9 activity is required for H_2_S production. Western blotting for Sch9 was used to verify that H_2_S production correlated with the concentration of Sch9 protein present in cells (lower panels, Figure 1A).

Decreased H_2_S production by *Δsch9* cells was also observed by measuring the reduction of methylene blue in different yeast strain backgrounds (BY4741 and BY4742) (Figure 1C). Measurement of intracellular H_2_S in these two yeast strains by using WSP-1 fluorescent showed the same trends (Figure 1D). Consistent with previous studies showing that CR enhanced H_2_S production in yeast [7], we observed a significant increase of H_2_S production in WT cell under CR (Figure 1E). However, similar to no restriction condition, H_2_S production was still significantly impaired in *Δsch9* cell even under CR (Figure 1E).

**Figure 2:**
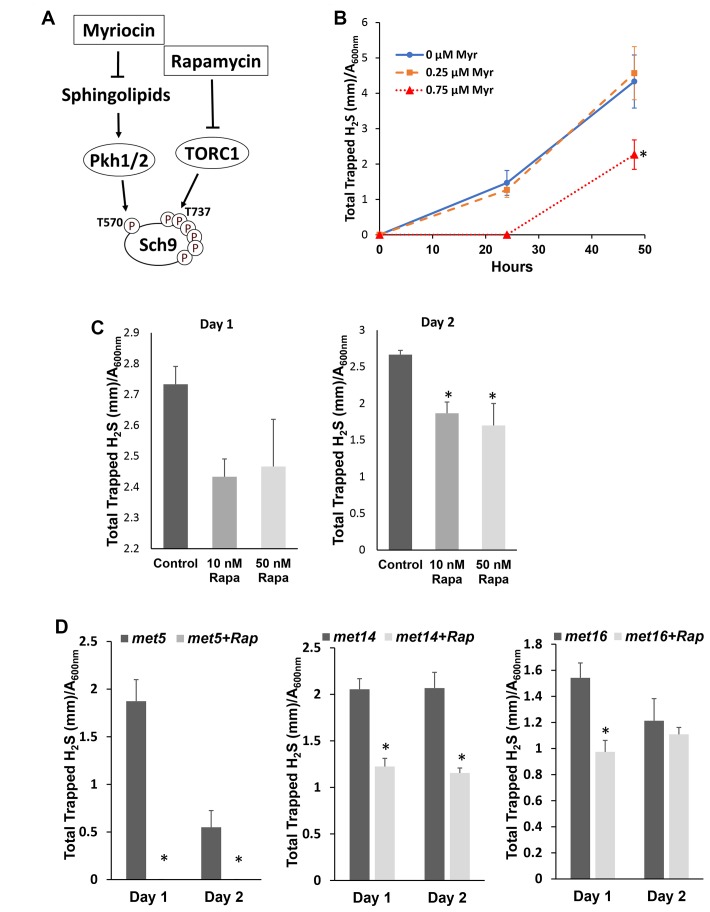
**Inhibiting Sch9 activity by rapamycin or myriocin treatment decreased H_2_S production.** (**A**) Diagram showing how rapamycin and myriocin inhibit Sch9 through two different signaling pathways. (**B**) H_2_S production by BY4741 was monitored by using lead acetate strips at 24 or 48 hours after inoculation into YPD medium containing the indicated concentrations of myriocin. (**C** and **D**) H_2_S production by BY4741 or sulfur assimilatory mutants was monitored by using lead acetate strips which were replaced every 24 hours after the indicated concentrations of rapamycin were added into overnight culture of YPD (* p<0.05 compared to control).

To investigate if phosphorylation of Sch9, which correlates with its kinase activity and is required for H_2_S production, two inhibitors, Myriocin and Rapamycin, that indirectly lower Sch9 activity were used to inhibit the phosphorylation of Sch9 at the activation loop and the hydrophobic motif, respectively [19] (Figure 2A). Treatment with myriocin at 0.75 μM, but not 0.25 μM, inhibited H2S production (Figure 2B). Similarly, Rapamycin treatments at 10 or 50 nM also resulted in decreased H2S production (Figure 2C). These data indicate that the phosphorylation of Sch9 at the both activation loop and the hydrophobic motifs are required to the regulate H_2_S production.

Unlike mammalian cells, yeast cells convert extracellular sulfate to sulfide through the sulfur assimilatory pathway with enzymes encoded by MET14, MET16, and MET5/10 in addition to conserved the TSP pathway [7]. To verify if the TSP pathway is involved in decreased H_2_S production by mTORC1-Sch9 inhibition in yeast, we monitored the effect of rapamycin in H_2_S production by MET14, MET16 and MET5 mutants. As shown in Figure 2D, all three mutants have decreased H_2_S production upon mTORC1 inhibition by rapamycin, suggesting that interfering with the sulfur assimilatory pathway does not change the inhibitory role of rapamycin in H_2_S production and the TSP pathway is likely involved.

### The role of mTORC1-Sch9 on the production of hydrogen sulfide is not caused by the alteration of methionine metabolism

**Figure 3:**
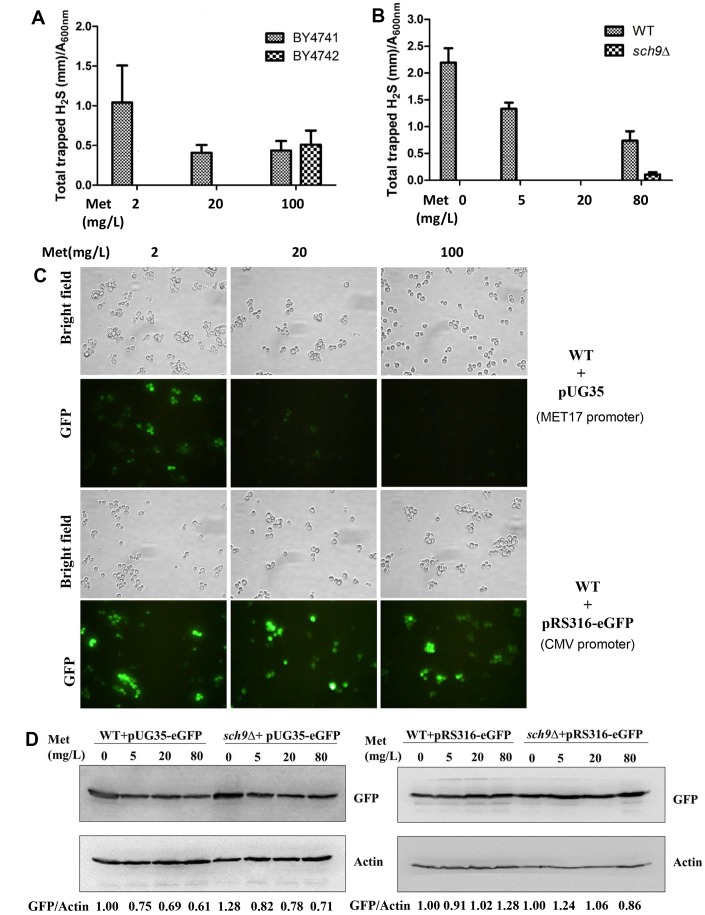
**Decreased H_2_S production in *Δsch9* cells is not due to methionine restriction.** (**A** and **B**) H_2_S production was monitored 24 hours after inoculation of BY4741 and BY4742 cells or WT and *Δsch9* cells (TB50a background) in the presence of indicated concentration of methionine in the SDC medium by using lead acetate strips. (**C**) Fluorescence microscopy of eGFP expression in BY4741 cell transformed with either pUG35-eGFP (with *MET7* promoter) or pRS316-eGFP (without *MET7* promoter). (**D**) Immunoblot analysis of GFP expression in WT and *Δsch9* cells (TB50a background) with actin as loading control. Cells were transformed with either pUG35-eGFP (with *MET7* promoter) or pRS316-eGFP (without *MET7* promoter). The ratios of GFP to Actin are quantified by ImageJ and indicated below the lower panels.

The mTORC1 pathway plays an important role in regulating cell growth in response to amino acid availability [19, 20]. Additionally, methionine metabolism contributes to H_2_S production through TSP pathway and methionine restriction extends lifespan from yeasts to humans [3, 8]. Therefore, we asked if there is a lowered free methionine pool in *Δsch9* cell which may contribute to the decreased H_2_S production. The effects of methionine on H_2_S production were investigated in BY4741, a strain with a defective *MET15* gene which prevents the synthesis of methionine from sulfate in the medium, and BY4742 with functional *MET15*. BY4741 cells produced more H_2_S in the present of 2 mg/L methionine than in the present of 20 mg/L methionine while BY4742 cell only produced barely detectable H_2_S at both conditions (Figure 3A). This suggests that methionine restriction indeed contributes to H_2_S production. It is worth to noting that when the methionine concentration in the medium was increased to 100 mg/L, H_2_S production increased in BY4742 cells. This may be due to the extreme abundance of substrates for H_2_S production. Similarly, in a different yeast background TB50a, decreasing the methionine concentration in the medium from 20 mg/L to 5 mg/L or 0 gave rise to significant H_2_S production while *Δsch9* cell remained defective in H_2_S production under those conditions (Figure 3B). Also, increasing methionine to 80 mg/L partially recovered H_2_S production by *Δsch9* cell probably due to the extreme abundance of substrates.

To investigate if there is a high level of intracellular methionine to inhibit H_2_S production by *Δsch9* cell, a methionine probe plasmid pUG35-eGFP was constructed by putting eGFP expression under *MET17* promoter which efficiency is inhibited by high concentration of intracellular methionine [21]. Indeed, the expression of eGFP protein by pUG35-eGFP was inhibited by increasing methionine concentration of medium (Figure 3C upper and Figure 3D left panels), while eGFP level was not altered by exogenous methionine in the absence of the *MET17* promoter (Figure 3C lower and Figure 3D right panels) indicating that the altered eGFP levels are not due to the degradation of the protein. However, when pUG35-eGFP was transformed into *Δsch9* cell the expression of eGFP was similar to that when the probe was present in WT cells (Figure 3D left panels), indicating that the intracellular level of methionine in *Δsch9* cell is not higher than that in WT cells and does not contribute to the decreased H_2_S production.

### mTORC1-Sch9 regulates H_2_S production by regulating cysteine metabolism

**Figure 4:**
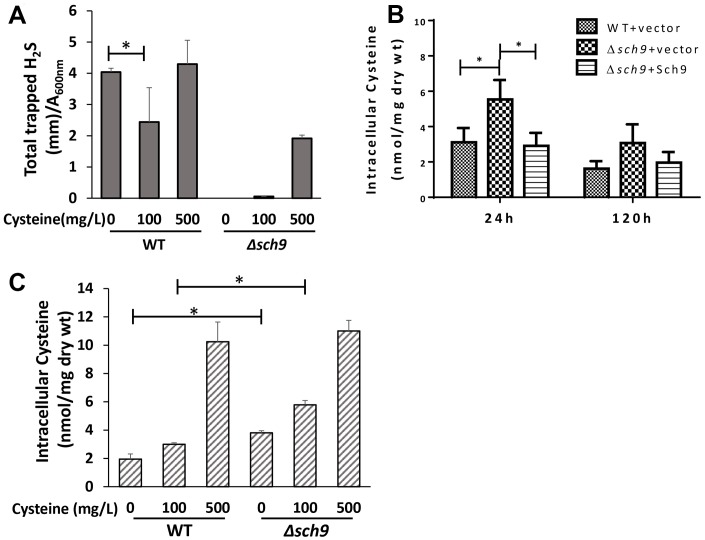
**Deletion of *SCH9* increases intracellular cysteine which regulates H_2_S production.** (**A**) H_2_S production was recorded 48 hours after inoculation of WT and *Δsch9* TB50a cells in the absence of methionine and in the presence of the indicated concentration of cysteine in the SDC medium by using lead acetate strips. (**B**) Intracellular levels of cysteine in WT and *Δsch9* cells in the TB50a background transformed with pRS316-*SCH9* or empty vector were measured by acid ninhydrin reagent. (**C**) Intracellular levels of cysteine in WT and *Δsch9* TB50a cells were measured by acid ninhydrin reagent in the absence of methionine and in the presence of the indicated concentration of cysteine in the medium. (* p<0.05).

Cysteine is another sulfur-containing amino acid whose metabolism is closely related to H_2_S production [3]. To verify that an altered intracellular level of cysteine is involved in the regulation of H_2_S production by mTORC1-Sch9, we first investigated how H_2_S production is affected by different levels of cysteine supplementation. Adding 100 mg/L cysteine to SDC medium lacking methionine significantly decreased H_2_S production in WT TB50a cell (Figure 4A). And increasing cysteine concentration to 500 mg/L restored H_2_S production likely due to substrate abundance (Figure 4A). Deletion of *SCH9* caused significant inhibition of H_2_S production under cysteine limited or over-supplied conditions (Figure 4A). These data suggest that the inhibition of mTORC1-Sch9 renders H_2_S production less sensitive to exogenous cysteine, probably due to increased endogenous cysteine. Indeed, unlike the intracellular methionine level which was not changed in *Δsch9* cell (Figure 3D), the intracellular cysteine level was ~50% higher in *Δsch9* cell than WT cell (Figure 4B). And when a functional *SCH9* gene was added back to mutant cells the intracellular cysteine level decreased (Figure 4B), showing that Sch9 regulates intracellular cysteine metabolism. When the level of exogenous cysteine was 100 mg/L, the intracellular cysteine level was still higher in *Δsch9* cells than WT cells until the level of exogenous cysteine reached 500 mg/L (Figure 4C). Together, these data indicate that the decreased H_2_S production by mTORC1-Sch9 inhibition is most likely due to an increase in the level of intracellular cysteine.

### mTORC1-Sch9 regulates H_2_S production via transsulfuration pathway

**Figure 5:**
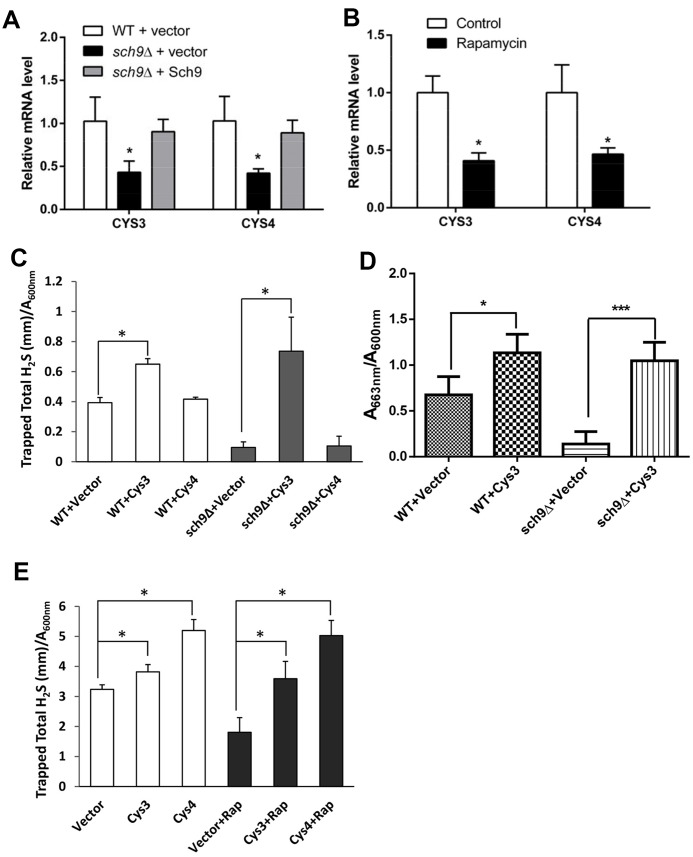
**Transsulfuration enzymes are involved in the H_2_S production regulation by mTORC1-Sch9.** (**A** and **B**) RT-qPCR analysis of *CYS3* and *CYS4* mRNA expression in TB50a cells in the presence or absence of Sch9 (**A**) or Rapamycin (**B**). (**C** and **D**) H_2_S production monitored by lead acetate strips (**C**) in WT and *Δsch9* BY4741 cells or methylene blue assays (**D**) in WT and *Δsch9* TB50a cells overexpressing *CYS3* or *CYS4* or with empty vector. (**E**) H_2_S production monitored by lead acetate strips in WT BY4741 cells overexpressing *CYS3* or *CYS4* or with empty vector with or without rapamycin treatment. (* p<0.05; *** p<0.005).

Increased intracellular cysteine usually inhibits the expression of enzymes in the transsulfuration pathway that required for H_2_S production [7]. To investigate if the expression of the transsulfuration enzymes is altered in *Δsch9* cell, the mRNA levels of *CYS3* and *CYS4* which encodes Cystathionine gamma-lyase and Cystathionine beta-synthase respectively in yeast were compared in *Δsch9* and WT cells. Indeed, the mRNA levels of both *CYS3* and *CYS4* decreased to about 50% in *Δsch9* cell compared to them in WT cell. And it can be reversed by adding *SCH9* back to mutant cells (Figure 5A). Similarly, inhibiting mTORC1-Sch9 by rapamycin also decreased the expression of both *CYS3* and *CYS4* (Figure 5B). These data suggest that inhibiting mTORC1-Sch9 which increases intracellular cysteine level does down-regulate transsulfuration pathway.

To verify the role of Cys3 and Cys4 in H_2_S production regulation by mTORC1-Sch9, *CYS3* or *CYS4* was overexpressed in *Δsch9* or WT cells treated with rapamycin and H_2_S production was monitored (Figure 5C to 5E). Overexpressing *CYS3* significantly increased H_2_S production by WT cell. While *Δsch9* cell with empty vector produced little H_2_S, overexpressing *CYS3* restored H_2_S production to a level similar to WT cells overexpressing *CYS3* (Figure 5C and 5D). Similarly, overexpressing *CYS3* also restored H_2_S production in rapamycin treated WT cell (Figure 5E). However, although overexpressing *CYS4* restored H_2_S production in rapamycin treated WT cell, it did not restore H_2_S production in *Δsch9* cell (Figure 5C). These data support the hypothesis that the *CYS3* and *CYS4* genes in the transsulfuration pathway mediate H_2_S production and are regulated by the mTORC1-Sch9 pathway.

### Inhibiting mTORC1 reduces H_2_S production and expression of transsulfuration pathway enzymes in human cells.

**Figure 6:**
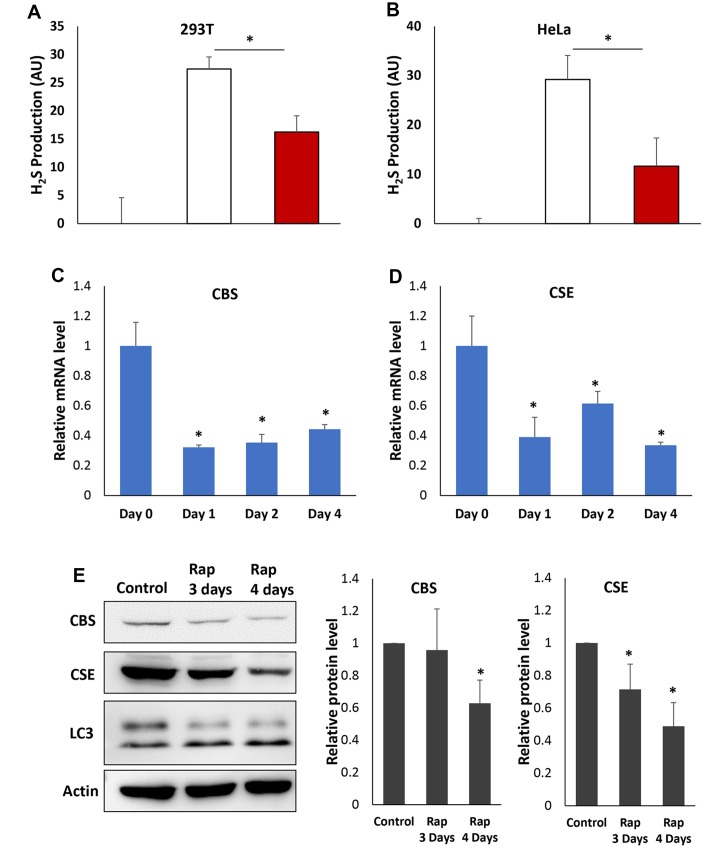
**Rapamycin inhibits H_2_S production and expression of CBS and CGL in human cells.** (**A** and **B**) H_2_S production was monitored in the presence or absence of Cys, PLP and rapamycin as indicated in 293T (**A**) or HeLa (**B**) cell. (**C** and **D**) Relative mRNA levels of CBS (**C**) or CGL (**D**) in HeLa cell treated with rapamycin for indicated times. Expression levels of β-actin mRNA were used as internal controls. (**E**). Western-blotting detection of CBS and CGL protein in HeLa cell treated with rapamycin for indicated times (Left). LC3 was also monitored to verify that the autophagy was induced by rapamycin. Quantification of CBS and CGL protein levels was based on Western blots and normalized to respective β-actin levels (Middle and Right). (* p<0.05).

The anti-aging effects of H_2_S production mediated by transsulfuration pathway and regulated by mTORC1 are evolutionary conserved from yeast to mammals [7, 11–13]. To assess if inhibiting mTORC1 also interferes with H_2_S production in mammalian cells, we examined human 293T and HeLa cells. To measure H_2_S production in cultured 293T and HeLa cells by using the lead acetate strip assay the growth medium was supplemented with the CGL/CBS substrates Cys and cofactor pyridoxal-5′-phosphate (PLP). The supplementation of Cys/PLP decreased the cell viability (Supplementary Figure 1), but significantly increased H_2_S production. With these assay conditions rapamycin treated cells have decreased the cell death induced by Cys/PLP and higher cell density (Supplementary Figure 1). However, less H_2_S is produced in both 293T and HeLa cells with rapamycin treatments (Figure 6A and 6B), similar to what we observed in yeast cells (Figure 2D). These data suggest that inhibiting mTORC1 in mammalian cells may also decrease H_2_S production.

To determine if reduction of H_2_S by inhibition of mTORC1 activity in mammalian cells is also accompanied by down-regulation of transsulfuration pathway enzymes, the expression of CGL and CBS was monitored by RT-qPCR in HeLa cell with or without rapamycin treatments for 1 to 4 days. Similar to what we observed in yeast cells (Figure 5B), rapamycin treatment reduced the mRNA level of both CGL and CBS significantly (Figure 6C and 6D). Consistent with mRNA levels, CGL and CBS protein levels were significantly decreased after 3 to 4 days of rapamycin treatments (Figure 6E), indicating that the expression of both transsulfuration pathway enzymes is reduced upon mTORC1 inhibition in HeLa cell.

## DISCUSSION

Due to the important roles of the mTORC1 and H_2_S signaling pathways in aging and longevity, we wanted to investigate if the widely studied anti-aging effect of mTORC1 is partially mediated by endogenous H_2_S production. Surprisingly, by monitoring the amount of H_2_S released from yeast cells and its intracellular level in different strain backgrounds, we found that H_2_S production was reduced when the mTORC1-Sch9 signal transduction pathway was inhibited. These observations are unexpected since both increased H_2_S production and lower mTORC1-Sch9 activity are beneficial for lifespan. The facts that DR lowers activity of the mTORC1-Sch9 pathway and enhances H_2_S production are also unexpected and suggest involvement of novel lifespan enhancing mechanisms. A plausible explanation is that the lifespan extension mediated by direct mTORC1-Sch9 inhibition under normal nutrition conditions does not require H_2_S. Conversely, H_2_S probably benefits lifespan through down-regulating mTORC1-Sch9. Thus further studies are required to investigate if H_2_S regulates mTORC1-Sch9 signaling and, if it does, then what is the mechanism.

Based on the unexpected observation of H_2_S production being down-regulated by mTORC1-Sch9 inhibition, we asked what mechanism is behind this phenomenon. Sulfur amino acid metabolism is likely involved since it is regulated by mTORC1 and contributes to endogenous H_2_S production. Indeed, H_2_S production is controlled by both methionine and cysteine levels [3], and only the cysteine level is enhanced by mTORC1-Sch9 inhibition (Figures 3 and 4), suggesting that an alteration of the cysteine level is involved in regulation of H_2_S production by mTORC1-Sch9. It is not surprising that inhibiting mTORC1-Sch9 increase intracellular cysteine level since many studies have shown that mTORC1-Sch9 inhibition enhances autophagy and decreases protein synthesis through its downstream factors and both processes may contribute to cysteine accumulation [22].

**Figure 7:**
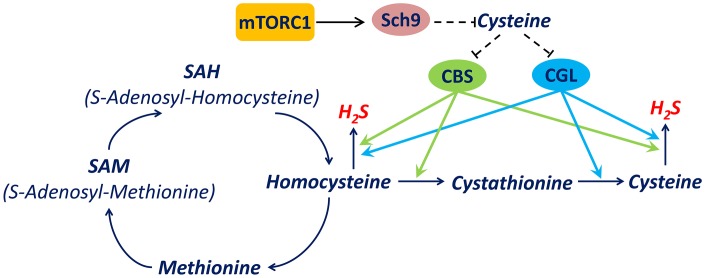
**A mechanism by which mTORC1-Sch9 regulates H_2_S production via transsulfuration pathway.** mTORC1-Sch9 controls the intracellular level of cysteine which is one of substrates for endogenous H_2_S production. On the other hand, cysteine regulates the expression of key transsulfuration pathway enzymes CBS and CGL which catalyze H_2_S production from homocysteine or cysteine. CBS is encoded by *CBS* in human and *CYS4* in Saccharomyces cerevisiae while CGL is encoded by *CTH* in human and *CYS3* in *Saccharomyces cerevisiae*. Dash lines indicate indirect regulations.

While cysteine is the substrate for H_2_S production and large increases in intracellular cysteine promote H_2_S production, it has been demonstrated that moderate increases in intracellular cysteine decrease H_2_S production by inhibiting expression of the transsulfuration pathway enzymes including CBS and CGL (Figure 7) [23–25]. In this study, we observed the downregulation of CBS and CGL accompanied with cysteine elevation upon mTORC1-Sch9 inhibition, supporting the hypothesis that decreased CBS and CGL activity in response to cysteine elevation contributes to decreased H_2_S production. The restoration of H_2_S production by restoring CBS or CGL enzyme activity during mTORC1-Sch9 inhibition is consistent with this mechanism (Figure 5). Therefore, we established a mechanism in yeast by which mTORC1-Sch9 regulates H_2_S production through altering intracellular cysteine level and expression of CBS and CGL (Figure 7).

Additionally, our data indicate that inhibiting mTORC1-Sch9 down-regulates the transsulfuration pathway and reduces H_2_S production in ways that are conserved in yeast and human cells (Figure 7). It worth noting that an earlier study indicated that constitutively activating mTORC1 in mouse hepatocytes prevents the increase of CGL expression and H_2_S production by DR [7]. However, it is not clear how the transsulfuration pathway and cysteine metabolism are altered in different types of cells at different situations. And, as indicated by our data, a moderately increased cysteine level inhibited H_2_S production while higher cysteine levels increased H_2_S production (Figure 4A). Therefore, H_2_S production seems to be very sensitive to the extracellular or intracellular level of cysteine and published data may have been influenced by how much cysteine accumulated in the tissues under different nutrition conditions and genetic backgrounds.

Together, our study reveals crosstalk between mTORC1 and H_2_S signaling, two conserved pathways which play fundamental regulatory roles in aging of eukaryotic organisms. Further studies which elucidate how these two pathways collaborate in specific human cells and tissues will have broad implications for potential clinical applications.

## MATERIALS AND METHODS

### Yeast strains, plasmids, and media

The S. cerevisiae strains and plasmids used in this study are listed in Table 1 and Table 2. Strains were grown at 30 °C in YPD medium (1% yeast extract, 2% peptone, 2% glucose) or synthetic dextrose complete medium (SDC) which contains no cysteine [26]. Cells transformed with plasmids carrying *URA3* were grown in the SDC medium lacking uracil. For inducing the expression of DNA sequences inserted into pYES2, cells were grown in the galactose-inducing medium (2% glucose, carbon source of SDC medium, was replaced to 1% Galactose and 1% Sucrose).

**Table 1:** *S. cerevisiae* strains.

**Strain**	**Genotype**	**Source**
BY4741	*MATa his3-Δ1 leu2-Δ0 ura3-Δ0 met15-Δ0*	Lab stock
RCD398	BY4741 with *sch9Δ::KAN*	Gift from Dr. R. C. Dickson
BY4742	*MAT alpha his3-Δ1 leu2-Δ0 ura3-Δ0 lys2-Δ0*	Lab stock
RCD399	BY4742 with *sch9Δ::KAN*	Gift from Dr. R. C. Dickson
TB50a	*MATa trp1 his3 ura3 leu2 rme1*	[14]
TS120-2d	TB50a with *sch9Δ:: KanMX*	[14]
met5	*MATa his3-Δ1 leu2-Δ0 ura3-Δ0 met15-Δ0 met5::KAN*	From BY4741 deletion collection, Open Biosystems
met14	*MATa his3-Δ1 leu2-Δ0 ura3-Δ0 met15-Δ0 met14::KAN*	From BY4741 deletion collection, Open Biosystems
met16	*MATa his3-Δ1 leu2-Δ0 ura3-Δ0 met15-Δ0 met16::KAN*	From BY4741 deletion collection, Open Biosystems

**Table 2:** *S. cerevisiae* plasmids.

**Plasmid**	**Constructive information**	**Source**
pRS316	Single copy plasmid vector, yeast endogeous promoter	Lab stock
pRS316- *SCH9*	pRS316 with 3HA after initiator codon of *SCH9*	Lab stock
pRS316-eGFP	eGFP gene was cloned into pRS316 vector	This study
pUG35-eGFP	eGFP gene was cloned into pUG35 vector, *MET17* promoter	This study
pYES2-NTA	Multicopy plasmid vector, GAL promoter	Lab stock
pYES2-*CYS3*	*CYS3* gene from BY4741 was cloned into pYES2-NTA, GAL promoter.	This study
pYES2-*CYS4*	*CYS4* gene from BY4741 was cloned into pYES2-NTA, GAL promoter.	This study

### Protein extraction and western blotting

For protein extraction, trichloroacetic acid yeast cell extracts were prepared according to a method described previously [17, 27]. HeLa cell with indicated treatments were washed with PBS and lysed in Laemmli buffer. Standard SDS-PAGE and Western blotting protocols were performed with primary antibodies including polyclonal rabbit anti-Sch9 antibodies (1:2000, a gift from Dr. Robert C. Dickson of University of Kentucky), monoclonal mouse anti-GFP antibodies (1:5000, Zen BioScience, China), monoclonal mouse anti-actin (1:5000, Zen BioScience, China), polyclonal rabbit anti-LC3B (1:2000, Abcam, USA), monoclonal rabbit anti-CGL (1:2000, Abcam, USA) or monoclonal rabbit anti-CBS (1:2000, Abcam, USA). Secondary antibodies include alkaline phosphatase-linked anti-rabbit or anti-mouse IgG (1:2000, Zen BioScience, China).

### RNA extraction and RT-qPCR analysis

Total RNA was extracted from yeast cell or HeLa cell with RNAiso Plus (TaKaRa Bio, China) or Beyozol reagent (Beyotime) respectively. For yeast cell, cells (OD600nm of 5) were pretreated with 50 U lyticase at 30 °C for 30 min to increase extraction efficiency. Reverse transcription reactions were conducted using a PrimeScript RT reagent kit with gDNA eraser (Takara Bio, China). The primers for real-time quantitative PCR (RT-qPCR) are indicated in Table 3. RT-qPCR experiments were performed using SYBR Premix Ex Taq II (TaKaRa Bio, China) and Bio-Rad CFX manager RT-qPCR system. Data were collected and analyzed by Bio-Rad CFX manager software.

**Table 3:** Primers used for RT-PCR.

**#**	**Primer name**	**Oligonucleotide sequence**
1	*ACT1*-F	5′- CGTTCCAATTTACGCTGGTT -3′
2	*ACT1*-R	5′- AGCGGTTTGCATTTCTTGTT-3′
3	*CYS3*-F	5′- CCCAACCAACCCAACTT -3′
4	*CYS3*-R	5′-CAGGACACCGAGCACAA -3′
5	*CYS4*-F	5′- CGAAGGTGTCTTGGTGGGT -3′
6	*CYS4*-R	5′- CCTGATGGAATCTGGGAAT -3′
7	*CBS*-F	5′- GCGGCTGAAGAACGAAATCC -3′
8	*CBS*-R	5′- TGTCCAGCTTCCCATCACAC -3′
9	*CTH*-F	5′- CAGCATGAGTTGGTGAAGCG -3′
10	*CTH*-R	5′- GAAGCTCAGCAAGGCTTTCG -3′
11	*ACTB*-F	5′- CCTGGGCATGGAGTCCTGTG -3′
12	*ACTB*-R	5′- AGGGGCCGGACTCGTCATAC -3′

### H_2_S assay for yeast cell

Lead acetate strips for measuring H_2_S were pasted on the top of inner wall of 125 ml culture flasks containing 25 ml media. 1 L culture flasks containing 250 ml media were used for TB50a cells which do not intensively produce H_2_S. H_2_S reacts with the lead acetate on the strip, creating a darkened band. The length of the darkened band is proportional to the amount of H_2_S produced during fermentation [28, 29]. The stripes were replaced every day or remained for entire experimental periods as described.

The methylene blue assay described previously for H_2_S detection was also performed in centrifuge tubes [30]. 36ml cells at OD600nm of 0.05 were divided in two parts. One of them was added with 2ml medium to monitor growth rate as measured by the absorbance at 600 nm and the another was the addition of 2 ml methylene blue reaction mix (1 mg/ml methylene blue, 100 mM citric acid buffer at pH 4.5), reacting with H_2_S dissolved in medium. Methylene blue decolorization by H_2_S were monitored at 663 nm and normalized to biomass.

Intracellular free H_2_S levels were also determined using the H_2_S fluorescent probe WSP-1 (Cayman, USA) [31]. 1 OD of cells were harvested and washed with PBS, then incubated with 10 mM WSP-1 for 1 h at 30 °C in dark. After washed three times with PBS (2.7 mM KCl, 1.75 mM KH_2_PO_4_, 10 mM Na_2_HPO_4_ and 136.75 mM NaCl, pH 7.4), the pellet was resuspended in 1 ml PBS. 10 μl of cells was added to a microscope slide, and WSP-1 fluorescence was monitored using CEWEI LWD200-37FT fluorescence microscope (CEWEI, China). Alternatively, the fluorescence was measured at 465/515 nm excitation/ emission using an f4500 fluorescence spectrometer (Hitachi, Japan). The fluorescence intensity was normalized according to the OD value.

### H_2_S assay for cultured human cells

293T or HeLa cell was grown in DMEM (Invitrogen) supplemented with 10% FBS, 50 U/mL penicillin, and 50 μg/mL streptomycin with or without 250 nM rapamycin. To measure H_2_S production, growth media was supplemented with or without 10 mM Cys and 10 mM pyridoxal-5′-phosphate (PLP) and a lead acetate strip was placed above the media in a 25 ml cell culture flask incubated in a CO_2_ incubator at 37 °C for indicated time.

### Measurement of the Cys content

The extraction and estimation of cysteine content in yeast cells were done as described previously [32]. Cells grown in SDC-Ura or medium containing different concentrations of Cys were harvested and washed with PBS twice by centrifugation. Cell pellets were dried at 50 °C until constant weight was achieved. Dried cells were lysed in liquid nitrogen and then 1ml of 10% TCA was added to 200mg cell powder. The homogenates were centrifuged at 2800×g for 60min. Acid ninhydrin were added to the extract, and reaction mixture was kept in boiled water for 10 min. After fast cooling, the A560nm absorbance of the reaction mixture was measured. The amount of cysteine in each reaction was determined using a standard curve.

### Statistical analysis

All data from at least three independent experiments. Error bars are presented as averages ± SD. Statistical analysis and comparisons were performed using two-tailed, unpaired Student t tests.

## Supplementary Material

Supplementary Figures
