# Supplementary material for: mTORC1-Sch9 regulates hydrogen sulfide production through the transsulfuration pathway
Source: Aging (Albany NY). 2019 Oct 3;11(19):8418–32. doi: 10.18632/aging.102327 (PMC6814617; doi:10.18632/aging.102327)
Supplement: Supplementary Figures [file aging-11-102327-s001.pdf]

SUPPLEMENTARY FIGURE

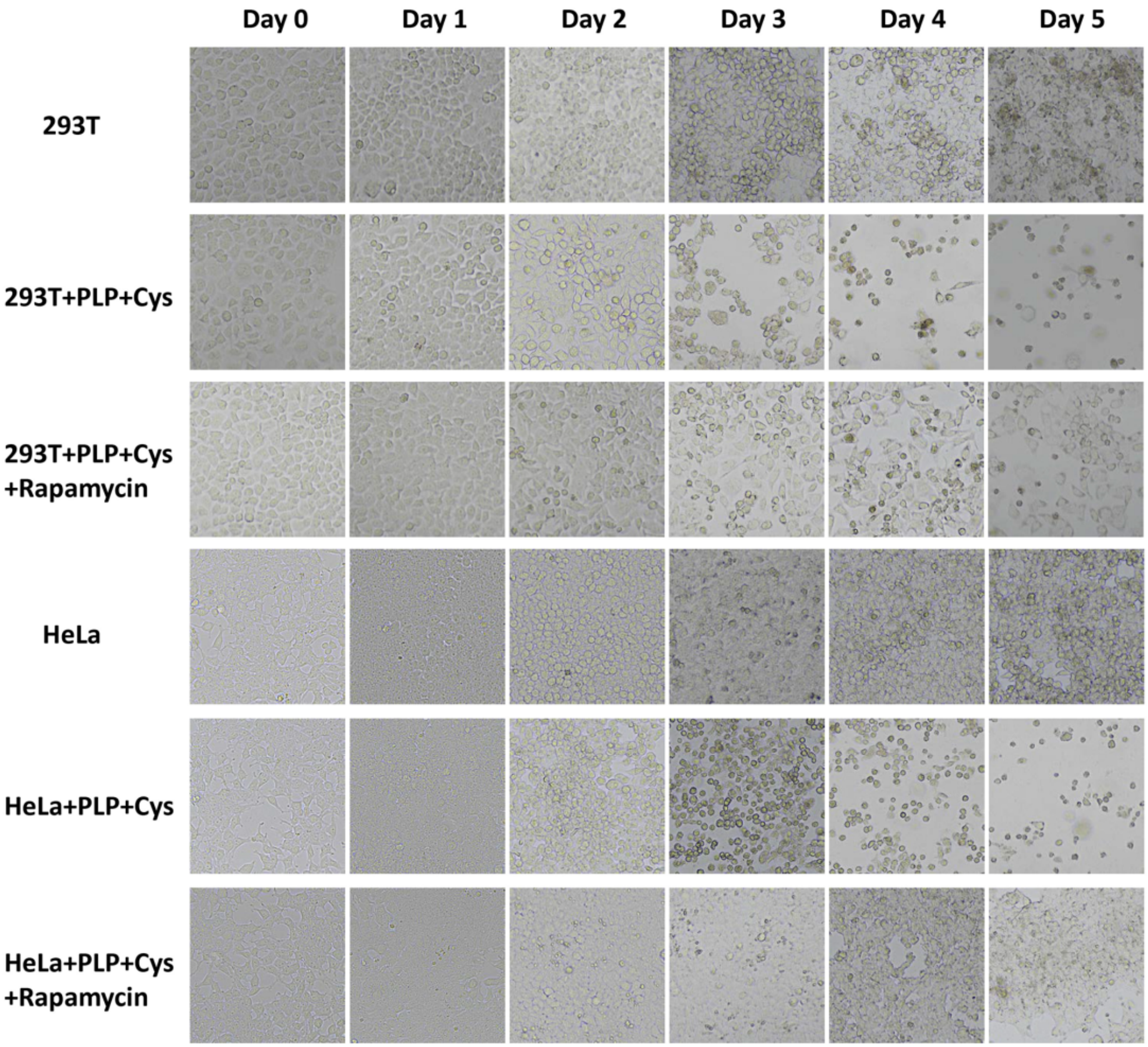

Supplementary Figure 1. Density of 293T and HeLa cells during the assay for H2S production. 293T or HeLa cells were cultured for 5 days with or without PLP, cysteine and rapamycin as indicated. Cell density was monitored every 24 hours using inverted microscope.
